# Knowledge about the impact of age on fertility: a brief review

**DOI:** 10.1080/03009734.2019.1707913

**Published:** 2020-01-22

**Authors:** Ilse Delbaere, Sarah Verbiest, Tanja Tydén

**Affiliations:** a Midwifery Education, VIVES University of Applied Sciences, Kortrijk, Belgium;; b Center for Maternal and Infant Health, School of Medicine, University of North Carolina at Chapel Hill, Chapel Hill, NC, USA;; c Department of Women’s and Children’s Heath, Akademiska Sjukhuset Uppsala University, Uppsala, Sweden

**Keywords:** Age, awareness, egg freezing, knowledge, fertility

## Abstract

Delayed childbearing is currently a major challenge in reproductive medicine as increased age has an important impact on successful conception, both in natural and in assisted reproduction. There is a lack of knowledge about the impact of age on fertility, even in highly educated populations. A number of initiatives have been taken to increase fertility awareness. Health care providers have been encouraged to talk with patients about their reproductive life plan (RLP) for almost a decade based on recommendations from the Centres for Disease Control and Prevention. This concept has been explored successfully in Swedish contraception counselling. A growing number of online interventions aim to raise fertility awareness. These websites or interactive tools provide relevant information for individuals and couples as they consider whether they want children, when they should have them, and how many they may wish to have. These interventions are important, because research depicts that knowledge helps people in their decision-making process. With new fertility preservations such as egg freezing now available, additional education is needed to be sure that women and couples are well informed about the cost and low success rates of this intervention.

## Introduction

Having children is valued very highly in all societies. Ninety percent of people in Western countries want children, generally between one and three (1–4). In higher-income countries, people have children because of ‘their contribution to life satisfaction’ of the couple, ‘development as a person’ in the parents, and ‘for giving and receiving love’. People in lower-income countries may also depend on children to contribute to the financial security of the family (1,5,6). The decision to have children or not may be moderated by individual, societal, and economic factors.

Interviews with professional women and men who did not have children found that they did not generally give much thought to their fertility, although there were variances between women and men. They believed that fertility problems could be addressed by assisted reproductive technologies (ART) or families could be formed through adoption. Postponed parenthood was described as an adaptation to societal changes and a contemporary lifestyle with many competing priorities (7).

Reproductive life planning is a simple concept that can be very complex. People who have experienced instability in their life, live with interpersonal violence, and/or live in poverty with limited options may not believe they have the ability to plan anything in their lives. Other people may feel ambivalent about ‘wanting’ a child for many reasons. Some people hold religious beliefs that run counter to the idea of planning. A person may wish to become a parent but not have a partner in their life. Reproductive plans often change over time due to life circumstances, including relationship changes. Family-friendly countries offer parental leave, subsidized childcare, and work places with flexible hours for parents so they can spend more time with their children as a way to create more equity for all people who may wish to become parents (8). Many countries, however, offer only limited support, which can make it more difficult for some people to balance family, professional, and financial needs.

This brief review aims to continue the conversation on this important topic by describing the impact of delayed parenthood on fertility and reviewing fertility awareness among university and medical students. This paper shares several successful interventions and offers recommendations for future work.

## Advanced maternal age and infertility

The number of women in high-income countries who have delayed childbearing has increased over the past decades, with the average age of having a first child being 30 years in Europe (9). There are a variety of reasons for this, including investment in education, development of a professional career, and/or difficulties in finding the right partner. Delayed childbearing is more common among women with higher education. As a consequence of delaying parenthood, there has been an increase in the number of women experiencing problems in getting pregnant, as well as higher risks for pregnancy and childbirth complications.

When a woman is younger than 30, she has an 85% chance to conceive within 1 year. At the age of 30, there is a 75% chance to conceive in the first 12 months. This chance declines to 66% at the age of 35 and 44% at the age of 40. This is due to the effect of aging on the ovary and eggs. Furthermore, older women are more likely to experience a miscarriage than younger women (27% of pregnancies end in a miscarriage at age 40 compared to 16% at age 30 or younger) (10). Advanced maternal age is associated with prolonged time to conceive, and postponed parenthood may affect the desired family size. Using a computer simulation programme, Habbema et al. calculated the recommended age to start a family for women, depending on the number of children they wanted and to what extent women were prepared to undergo fertility treatment. The model predicts that if a couple wanted a 90% chance to realize their ideal family without *in vitro* fertilization (IVF), couples with a desire for a one-child family should start at the latest at age 32 of the female partner. When a two-child family is desired they should start when the woman is 27, and when couples want three children they should start at age 23 (11). A computer simulation was also used in research by Leridon (10) to assess whether assisted reproduction could compensate for the effect of age on fertility. Unfortunately, this was not the case (10).

## Fertility knowledge among men and women of fertile age, with focus on university students

In 2004, Lampic, Skoog Svanberg, and Tydén started to investigate Swedish university students’ attitudes about having children and their knowledge about fertility (1,6,12). Although the majority of women wished to have two or three children, six out of ten said they would consider having an abortion if confronted with an unplanned pregnancy ‘right now’. Important prerequisites for women’s decision to have children were to be sufficiently mature, have a stable partner to share parenthood with, have completed studies, and have financial security. Women worried about problems with combining work and having children, including being less competitive in the Labour market. Slightly more than half of the women wanted to have their last child between ages 35 and 44 years. One-fourth of doctoral students overestimated a woman’s ability to become pregnant between 35 and 40 years of age, and about half had overly optimistic perceptions of the chances to have a baby by means of IVF. Women were less certain than men of being able to achieve the desired number of children (1,6,12). Sobotka commented on these studies, highlighting that it is a challenge for health care providers to educate university students about fertility issues when they want to postpone parenthood (13). The importance of this education was highlighted, and engaging men is important as male infertility is on the rise (14). Finding a partner to share parenthood with can be hard for women who intend to invest time in their own career (15,16).

The Swedish Fertility Awareness Questionnaire has been used in university populations in many countries with similar findings as in Sweden (17). The Swedish surveys investigated fertility knowledge with eight questions:

1. At what age are women the most fertile?
2. At what age is there a *slight* decrease in women’s ability to become pregnant?
3. At what age is there a *marked* decrease in women’s ability to become pregnant?
4. A young woman (<25 years) and a man have unprotected intercourse at the time of ovulation—how large is the chance that she will then become pregnant?
5. A woman and a man regularly have unprotected intercourse during a period of 1 year—how large is the chance that she will become pregnant if she is 25–30 years old?
6. How large is the chance that she will become pregnant if she is 35–40 years old?
7. How many couples in Sweden are involuntarily childless?
8. For couples that undergo treatment with IVF, what is their chance, on average, of having a child?

To summarize in brief: men and women had misconceptions about age and fertility and overestimated the success rate of having a child through IVF. In the case of infertility, women were more likely than men to consider adoption, but both genders were much more likely to choose IVF over adoption, indicating an importance of genetic parenthood.

As a consequence of these misconceptions, young men and women of reproductive age would benefit from evidence-based education on fertility issues to help them make informed decisions regarding reproductive planning. Two recent reviews investigating knowledge about age-related fertility decline concluded that campaigns about age and fertility should be targeted both to people of reproductive age and to health care providers. Interventions and campaigns are warranted, especially those targeting men and people with low education. They should be customized to meet individual needs (18).

## Fertility awareness among health care providers and medical students

Not only is there a lack of fertility awareness in the general population of university students, there is a lack of knowledge among medical students and health care providers as well. Medical students’ intentions for future parenthood and their knowledge about fertility have been investigated in several studies. Yu et al. used some of the Swedish fertility questions in a study of US obstetrics and gynaecology (OB/GYN) residents. Nearly half of the residents were misinformed about fertility decline, and three out of four overestimated the effectiveness of IVF treatment. Interestingly, 80% believed that they should initiate discussions about age-related fertility decline with patients. The findings indicated a need for improved education on age-related fertility decline in OB/GYN residency programmes (19).

In an Austrian study, fertility awareness was compared between medical students and non-medical students (20). Although medical students had a higher awareness of the impact of age on fertility than non-medical students, the general knowledge of fertility was low also among medical students. Similar results have been found in American, Serbian, Ukrainian, and Saudi Arabian samples (21–25). More than 95% of Serbian students, regardless of gender, wanted to have children; most indicated three as the desired number of children. However, their knowledge about fertility was inaccurate as well (22). Among Ukrainian students, six out of ten respondents reported there was a pronounced decline in female fertility after the age of 45 years (23). Szücs found better results in a sample of Hungarian, Serbian, and Romanian female university students in health sciences. They had significantly more knowledge about the menstrual cycle compared to other university students (26). Authors across these studies call for more attention to reproductive health and fertility awareness in medical education. Current research indicates that future health care providers are not sufficiently prepared to address fertility-related issues with their clients.

## Clinical setting-based interventions to increase fertility knowledge

As more women postpone childbearing until their late 30s and early 40s, it is clear that women of all ages would benefit from a better understanding of the biomedical limitations and other issues related to reproductive health that may help or hinder their reproductive plans. Working with young adults to create a reproductive life plan is one strategy (27). A reproductive life plan (RLP) provides an opportunity for both men and women to reflect upon their interest and hopes for becoming parents and to receive services that align with their wishes, including contraceptives, health education, and/or preconception care. A person’s immediate plan for becoming pregnant or not can also influence clinical care (e.g. prescription of certain medications). All people should receive care for conditions that could reduce fertility, and preventive health care services.

In an article on clinical content of preconception care, Jack et al. offered the following recommendation:

Routine health promotion activities for all women of reproductive age should begin with screening women for their intentions to become or not become pregnant in the short- and long-term and their risk of conceiving (whether intended or not). Providers should encourage patients (women, men, and couples) to consider a reproductive life plan and educate patients about how their reproductive life plan impacts contraceptive and medical decision-making. Every woman of reproductive age should receive information and counselling about all forms of contraception and the use of emergency contraception that is consistent with their reproductive life plan and risk of pregnancy. (28)

We believe that it is relatively easy to follow these recommendations when women seek family planning counselling as long as appointment times are long enough to allow for conversation.

In Sweden, midwives are responsible for approximately 90% of contraceptive counselling, and the counselling is free of charge. RLP counselling has been evaluated in studies conducted in Sweden (29–32), in the US among physicians (33–35), and in Iran at a health centre for women receiving maternal and child health care (36). The evaluations pointed out that while RLP counselling had no effect on effective contraception use, knowledge of fertility and awareness of preconception health increased (30–32). After the counselling, a higher proportion of the male Swedish participants expressed the goal to have children in their life (31). The expressed age for having the last child among female university students was lowered (30). These effects were not found in Iran (36). Participants experienced RLP counselling as predominantly positive (30–33,36).

Mittal et al. used the RLP counselling in a small pilot study with women with chronic diseases. Understanding the risks of pregnancy associated with diabetes, hypertension, and obesity increased, as did knowledge about how a reproductive plan could help them think about future decisions (34).

Stern and Robbins explored administrators’ and health care providers’ experience of using RLP counselling in clinical settings (29,37). Nearly 60% of health centres reported having written protocols for RLP, and 87% of providers reported frequent use of the RLP during family planning counselling with female clients (37). Midwives found The RLP counselling to be a positive experience, in that the counselling was rewarding and easy. They were aware of the importance of approaching this topic carefully and thoughtfully as there are many individual and societal factors that influence a person’s ability to create an RLP (29).

One key lesson from the Swedish experience is that the support of senior midwife leaders and clinic administrators is important for advancing and sustaining RLP counselling (38). The interest in RLP counselling is growing across Sweden, with many coordinating midwives asking the researchers for training and information.

In Denmark, at the University Hospital in Copenhagen, a fertility assessment and counselling (FAC) clinic was begun in 2011 as an addition to family planning clinics, which were established in the 1970s. Delayed childbearing, low fertility rates, and increasing use of social egg freezing prompted the introduction of this new service. The state-funded FAC clinic offered free counselling using a risk assessment score sheet which included measurement of anti-Müllerian hormone, ovarian and pelvic sonography in women, and sperm analyses in men. A fertility expert provided a physical exam and 30-min consultation to all patients. A campaign was launched to make women and men aware of this new clinic (39). After 1 year, a qualitative study explored women’s perceptions about the extent to which the fertility assessment intervention influenced their decision and choices in family planning. Most women indicated that their knowledge on fertility-related issues increased after they attended the counselling. This knowledge helped them to make informed decisions or to seek fertility treatment. Interestingly, older women who became aware of their closing fertile window stated that they felt more ready go ahead and get pregnant and no longer ruminating on pros and cons of having children (16). After 2 years the first 570 women who received care responded to an email questionnaire regarding subsequent pregnancies. The mean age was 35 years at inclusion, and 38% were single. Most of them (68%) tried to conceive within 2 years after attending the FAC clinic; three-quarters of these had achieved a pregnancy, 21% were still trying, and 5.4% had given up (40). Two-thirds (65%) of the women who had low risk scores conceived spontaneously within 12 months. The presence of at least one high risk score reduced the odds of achieving a pregnancy within 12 months by 75%. One-third of the pregnancies in the 2-year follow up were achieved by medically assisted reproduction. The researchers concluded that the FAC clinic concept was feasible and provided an important service to help support women in achieving their reproductive life plan (40). Future studies should evaluate the cost-effectiveness of the FAC clinic.

However, addressing pregnancy intention in a primary care setting may be perceived as implicit persuasion for some women, as has been showed in qualitative research on Latina and black women (41). Garbers et al. (42) conducted community-based participatory research to investigate the experience of pregnancy intention screening in community-based settings in black and Latina women aged 15–49. Three themes emerged from this research: personal agency, judgement and shame, and expertise versus authority. The community advisory board that was collected for this study recommends among others that health care providers should initiate pregnancy intention screening in a non-judgmental way and should be supportive of the agency of the patient. Also, body language and terminology used are important within the consultation.

## Online fertility education

Websites rather than doctors have been claimed to be the main source of fertility-related information for people aged 15–45 (5). Unfortunately, not all information provided by ‘Dr Google’ is trustworthy. There is still a need for easily accessed and attractive information from reliable sources (43). This is because online interventions seem to have a positive impact on health behaviours, and online information may thus be a cost-effective manner to disseminate fertility awareness (44–46).

There are several good online interventions that have been developed to increase fertility awareness. The Fertility Status Awareness Tool (FertiSTAT) by Bunting and Boivin, for example, can be used by couples or individuals to calculate the fertility status based on some questions (47). In the USA, a new online resource, the In Vitro Fertilization Success Estimator Tool, released by the Centres for Disease Control and Prevention helps people estimate the chance of having a live birth using IVF. Both patients and clinicians may use this tool to enhance counselling and communication (48). Additional resources and information related to ART success rates can also be consulted on the website.

Ekstrand et al. developed a website with RLP information. The website was translated into English, French, Arab, Greek, Spanish, and Somali to facilitate the use among women who could not read Swedish. A majority of midwives identified the quiz about fertility as a helpful tool (49). Midwives in primary health care testing this website found it useful and referred clients to read more on their own. One midwife noted that the tool ‘is easy to display, easy to understand, easy to recommend the woman to look into and read on her own’ (50).

An online intervention in the UK that provides fertility information to adolescents aged 16–18 years and university students aged 21–24 years significantly increased fertility knowledge for university students and also reduced the threat of infertility for university students and adolescents. Participation in the study was associated with an increase in feelings of anxiety but a decrease in physical stress reactions. Adolescents had more optimal fertility plans compared to emerging adults due to being younger (51).

Other online tools with the aim to increase fertility awareness and promote preconception health include:

- yourFertility.org.au (Australia): a comprehensive website with an interactive tool to calculate the best period to conceive, information on the impact of age, lifestyle, and weight on fertility and medical issues impacting fertility.
- nhs.uk/live-well/infertility (UK): an informative overview of causes, diagnosis, and treatment of infertility, but also of risk factors and how to preserve the fertility.
- MyFertilityChoices.com (Canada) provides information on fertility testing, preservation of fertility, treatment, and family-building options. There are decision-making resources to facilitate fertility choices. Personal stories can be shared on the website, and experts can be contacted with questions.
- Reproduktivlivsplan.se (Sweden) is an online version of the RLP counselling described above (30). People can indicate whether they want to have children or not, and, if they do, whether they want them within 1 year or later. As such, tailored information is provided according to the situation of the couple. Furthermore, information about fertility and lifestyle issues is provided, and visitors of the website can test their knowledge.
- ShowYourLoveToday.com (US) provides information to young adults about health and wellness, including information about fertility awareness.
- On a website from Finland, you can take a reproductive health test and a reproductive health quiz. It is offered in Finnish and English: https://repro.tamk.fi/app/select.
- Other tools are currently in development. In Belgium, results of a study on fertility awareness in a Flemish population encouraged the researchers to develop an interactive website to inform people about their reproduction. It can be consulted soon at www.Klaarvoorkinderen.be.

**Figure 1.**
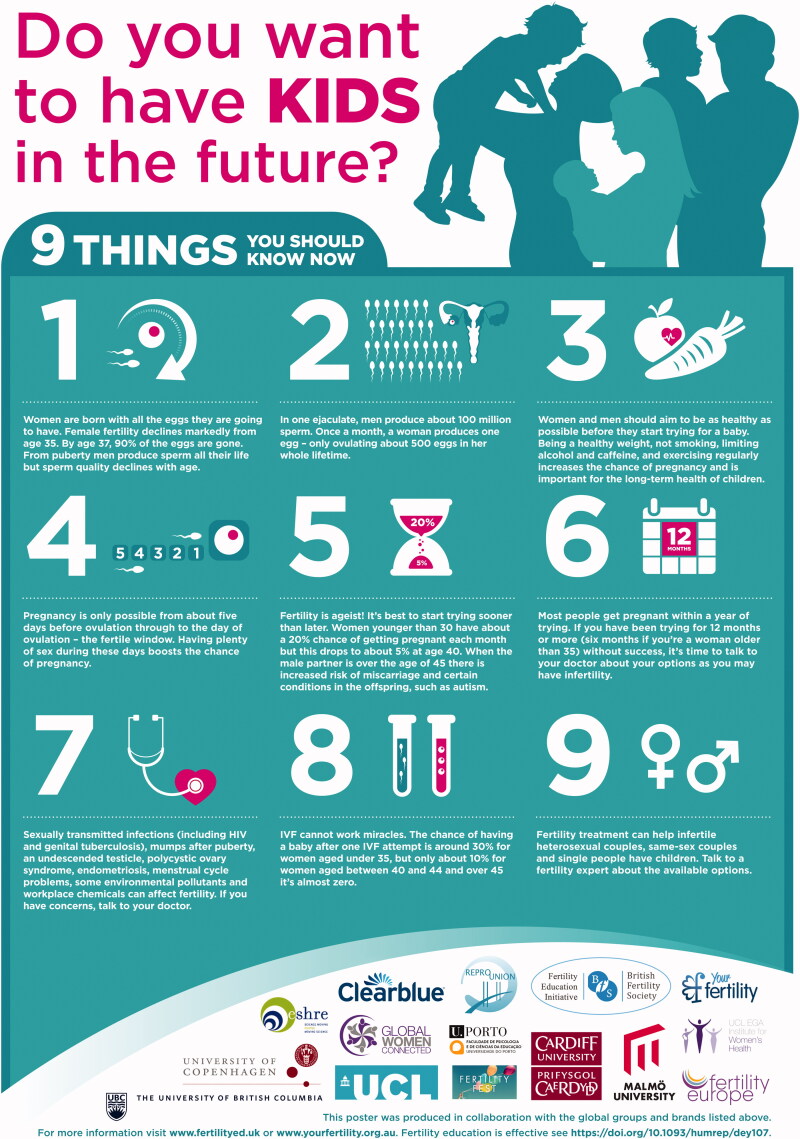
Fertility Education Poster. Source: Harper (52).

The recent European Congress on Preconception Health and Care in Denmark (September 2019) was a ‘fertile’ breeding ground for new ideas. Several researchers who designed some of the online tools described above worked together to develop a fertility education poster with very clear information for those who want to have children in the future (Figure 1) (52).

## Egg preservation

One option that women who wish to postpone pregnancy have is to freeze their eggs (oocyte banking)—at least for those who can afford it; egg preservation is costly and often not covered by health insurance plans. Some large companies are offering egg freezing as a fringe benefit for their employees. There are many emerging questions as to who benefits from postponing family creation. Is it the companies who will have hardworking employees without children? Is it the clinics who offer this technical reproductive method? Is it the women who can invest in career goals while trying to find a suitable partner? Is it the scientists who profit from the fees? In spite of the many unanswered questions, there is a rapidly growing interest in egg freezing that should be carefully monitored and examined over time.

One out of 10 female university students (average age 23 years) who attended a clinic for contraceptive counselling in Sweden in 2014 had considered freezing their eggs (53). In Italy, one-third of female university students had heard about the possibility of fertility preservation through egg freezing. Of these, 20% were in favour of egg freezing, and half of the women thought that the cost for this procedure should be borne entirely by the woman herself (54). Among women in the UK being counselled for age-related egg freezing, the average age was 36.7 years; this suggests that the women were not fully aware of the decline in egg quality from the age of 30 on (55).

A qualitative study with women on the waiting list for oocyte banking showed that these women were financially independent and lived in single-person urban households. They opted for oocyte banking because they wished to share parenthood with a future partner rather than becoming a single parent. Although women found the costs of the intervention high, they were willing to invest their money to increase their chances for shared parenthood (56). Baldwin and Culley found that women requesting egg freezing were generally satisfied with the treatment they received from clinics. However, they expressed a desire for more detailed information about potential outcomes from egg freezing (55).

While oocyte banking may be an option for women with a strong wish for a child in the future, the current technique is an unreliable insurance. If eggs are frozen before the age of 35, women have a 30–40% chance to conceive when there are 10 eggs available. However, research points out that women interested in elective egg freezing are often at the end of their reproductive life spans (late 30 s to early 40 s). These women want children, but have no partner with whom to build a family (57). Although the chances to have a child are rather low, the procedure is expensive (between 1500 €and 3000 €per cycle) and not affordable for everyone.

## Future directions

In light of this research, it is important to chart a careful and comprehensive course for the future. Education about human reproduction, including fertility, should be widely available beginning in adolescence. People need clear information about their reproductive health, including menstrual cycles and medical conditions and behaviours that can potentially compromise fertility. There are a number of new tools available to support this education. More research is needed to evaluate the efficacy of these tools, including any unintended consequences, as well as to design and test strategies for use in schools, universities and other settings.

Likewise, health care providers (including midwives and nurses) should receive education about fertility, both during their medical education and through continuing education opportunities. As IVF, oocyte banking, and other technologies continue to develop, providers need to be informed about the efficacy, costs, and success rates so as to provide accurate information to their patients.

Education and counselling around reproductive life planning and fertility should be approached carefully and thoughtfully, always thinking about the unique needs and situation of the client. Conversations on this topic may be upsetting and frustrating for people who may not be able to act on the advice given. Providers require training so they can approach these conversations without bias and in a way that supports each person’s needs and goals. The Family Planning National Training Centre (FPNTC) developed a Client-Centered Reproductive Goals & Counselling Flow Chart to improve health care providers’ ability to have client-centered conversations about fertility (58). Services to support reproductive goals, including access to contraceptive methods, preventive health care services, specialty care, relationship support, violence prevention, economic advancement, fertility monitoring, and fertility assistance, must be available to all people, not just those who can afford them.

Men must receive counselling and be engaged in conversations around fertility and parenthood. Research needs to be undertaken to listen to men on this topic and work with them to design messages and identify the best approach for having this conversation. Stories that only highlight the ‘miracle babies’ conceived and birthed by older couples may sell magazines but can also do harm by creating false expectations.

Society at large also plays a critical role in supporting new families so parents can care for their children but also have the opportunity to succeed in achieving their educational and career goals. While countries with family-friendly policies have taken many steps in this direction, ongoing public discussions and research are needed to identify additional strategies that can support gender equity and reproductive autonomy and support for all people. Moreover, all deserve to understand human reproduction, including how to manage their fertility. At the same time, providing this information can be very challenging as it can be perceived as judgmental and upsetting and must be offered within the context of many desires—educational and professional success, relationship with a trusted partner, a sense of readiness for the responsibility, and hoping to have a child/children in their lives. Further, not everyone has the resources, health, and opportunities to plan for the future. Efforts need to be made to not only provide information but to address these larger issues using an equity lens so that everyone can benefit and thrive.
